# GNSS Spoofer Localization with Counterfeit Clock Bias Observables in a Dynamic Scenario

**DOI:** 10.3390/s26196027

**Published:** 2026-09-23

**Authors:** Haoqing Li, Kyle O’Keefe

**Affiliations:** 1 School of Computer Science and Engineering, Constructor University, 28759 Bremen, Germany; 2 Constructor Labs, 28759 Bremen, Germany; 3 Department of Geomatics Engineering, University of Calgary, Calgary, AB T2N 1N4, Canada; kpgokeef@ucalgary.ca

**Keywords:** GNSS spoofing, dynamic spoofer localization, Kalman filtering, clock bias and drift

## Abstract

Global Navigation Satellite Systems (GNSSs) are widely used in daily life, and the popularity of GNSS-based applications has triggered concerns about the potential vulnerabilities of GNSS. One of the major threats is the spoofing signal, and localizing the spoofer enables the threat to be mitigated at its source. Most spoofer localization methods rely on a spatially distributed sensor network with accurate time synchronization requirements. Recent research has used a least-squares (LS) estimator to localize a static spoofer using clock bias or clock drift from a single antenna. In this paper, we propose and test an extension to a method recently published in the IEEE Sensors Journal that used an LS estimator to localize a spoofer using clock bias as an observation. Specifically, we propose to extend this work with an extended Kalman filter-based algorithm to locate a dynamic spoofer using clock-bias measurements obtained from a single antenna as observations. Therefore, dynamic spoofer tracking can be realized without deploying multiple spatially distributed receivers. We demonstrate the efficiency of the proposed method by comparing it with the static spoofer localization method using a properly designed simulation. The results show that the proposed method achieves better spoofer localization accuracy than the original LS method for static spoofer localization and can be applied to track a dynamic spoofer.

## 1. Introduction

Global Navigation Satellite System (GNSS)-based applications must deal with problems caused by various threats to GNSS signals. Spoofing is one of the major threats that severely affects the integrity and accuracy of GNSS-based applications. Spoofing of GNSS signals is the broadcast of false signals with the intent that the victim receiver will misinterpret them as authentic signals [1]. Usually, the spoofed signal is trying to misdirect the victim by broadcasting signals with different delays and Doppler frequencies that will cause the receiver to compute an erroneous position solution.

There are three general types of spoofers depending on how the spoofing signal is generated: meaconers, signal simulators, and receiver–repeaters [1,2,3]. A meaconer is a spoofer that retransmits the live GNSS signal it has received, possibly with amplification and additional delay, causing nearby receivers to lock onto the stronger spoofing signals rather than the authentic signal; a signal simulator replaces the live signal with a synthetic signal generated and transmitted locally; a receiver–repeater processes the received authentic GNSS signals first, and then retransmits locally generated signals to the victim receivers. This last type of spoofer is potentially the most dangerous, as it can start a spoofing attack with signals that resemble the authentic signal and then slowly amplify the spoofing signal and inject false position information.

Anti-spoofing techniques can be categorized into three types: spoofing detection, spoofing mitigation, and spoofer localization, and the metrics chosen to characterize spoofing influence in each method are different. For instance, in spoofing detection and mitigation, the metrics to characterize spoofing signals include Automatic Gain Control (AGC) [4], signal spatial correlation [5], Doppler shift [6], carrier-to-noise-density ratio (C/N0)-related measurements [7,8], Cross Ambiguity Function (CAF)-related metrics [9,10,11,12], pseudorange-based observations [13,14,15], carrier phase-based measurements [16,17] and a mixture of the above measurements [18]. On the other hand, the metrics in spoofer localization can be Received Signal Strength (RSS) [19], Angle of Arrival (AOA) [20], and Time-Difference-of-Arrival (TDOA) [21].

Most spoofer localization algorithms need more than one receiver [14,22,23]. Such methods generally require multiple spatially distributed receiver units. In particular, methods relying on measurements obtained at different receivers, especially time-related measurements such as TDOA, require high-precision time synchronization among the receivers. The use of multiple synchronized receivers also increases hardware and communication requirements, as well as the cost and complexity of system deployment. These practical limitations make a localization method using only a single antenna particularly attractive. Recently, single-receiver spoofer localization methods based on clock-related measurements were proposed in Refs. [24,25]. In particular, Ref. [24] showed that a stationary spoofer can be localized using counterfeit clock bias observables from a single receiver. In this paper, we assess the single-receiver spoofer localization technique in Ref. [24] and extend it to the case of a mobile spoofing source. In Ref. [24], a static spoofer is localized using counterfeit clock-bias observations collected by a single receiver along a known trajectory. The receiver positions and the corresponding counterfeit clock-bias observations establish the geometric relationship used to estimate the spoofer position. We believe this is a very promising method, and the purpose of this paper is to demonstrate that it can be extended to the case where the spoofing source is itself in motion by replacing the LS estimator in Ref. [24] with an extended Kalman filter [26]. The proposed method shares the same benefits as stated in Ref. [24], namely, simplified receiver hardware and reduced cost. In addition, it does not require high-precision synchronization among multiple spatially distributed receivers. Furthermore, because the proposed method considers prior information using a Kalman filter (KF), it provides higher spoofer localization accuracy than the LS-based algorithm in [24].

The remainder of this paper is organized as follows: Section 2 describes the observation model and localization algorithm; Section 3 presents simulation results replicating the results of Ref. [24] and extending them to the case of a moving spoofer. Section 4 concludes the paper and provides suggestions for future work.

## 2. Dynamic Spoofer Localization Method

### 2.1. Counterfeit Clock Bias Observable Model

We assume a fully spoofed scenario and use the notation in Ref. [24] to express the pseudorange from the receiver to the *j*-th counterfeit satellite at the *k*-th epoch as follows:

(1) $$\begin{array}{cc} \rho_{j}^{s}\left(k\right) & =r_{j}^{s}\left(k\right)-c\delta t_{j}^{s}\left(k\right)+I_{j}^{s}\left(k\right)+T_{j}^{s}\left(k\right)+c\delta t_{u}\left(k\right)\\ \; & +c\delta t_{d}^{s}\left(k\right)+L\left(k\right)+\epsilon \left(k\right) \end{array}$$

where $r_{j}^{s}\left(k\right)$ is the geometric distance from the *j*-th counterfeit satellite’s position $S_{j}^{s}\left(k\right)$ to the false position $p_{f}\left(k\right)$ that the spoofer wants the victim receiver to output. $c\delta t_{j}^{s}\left(k\right)$, $I_{j}^{s}\left(k\right)$, and $T_{j}^{s}\left(k\right)$ are the corresponding counterfeit satellite clock error, ionospheric delay, and tropospheric delay, respectively. $c\delta t_{u}\left(k\right)$ is the receiver clock bias, $c\delta t_{d}^{s}\left(k\right)$ is the equivalent sum of the spoofer’s clock bias, hardware delay, and deliberately induced time bias, and L(k) is the signal propagation delay from the spoofer antenna to the receiver antenna, given by $L\left(k\right)=\;∥p_{u}^{\mathrm{ECEF}}\left(k\right)-p_{s}^{\mathrm{ECEF}}\left(k\right)∥$, with $p_{u}^{\mathrm{ECEF}}\left(k\right)$ and $p_{s}^{\mathrm{ECEF}}\left(k\right)$ as the receiver and spoofer positions, and ϵ represents the noise. For simplicity, we define $p_{u}^{\mathrm{ECEF}}\left(k\right)$ and $p_{s}^{\mathrm{ECEF}}\left(k\right)$ as $p_{u}\left(k\right)$ and $p_{s}\left(k\right)$ in the remainder of the paper.

After correcting the satellite clock error, the ionospheric delay, and the tropospheric delay of each spoofing signal’s pseudorange, the counterfeit clock bias $B_{f}\left(k\right)$ can be modeled as

(2) $$\begin{array}{c} B_{f}\left(k\right)=c\delta t_{u}\left(k\right)+c\delta t_{d}^{s}\left(k\right)+L\left(k\right)+\xi \left(k\right) \end{array}$$

where ξ(k) represents the uncertainty of the estimated counterfeit clock bias.

### 2.2. Extended Kalman Filter-Based Estimator

With the counterfeit clock bias $B_{f}\left(k\right)$ as an observation, we assume a constant velocity model for the spoofer and localize it using an extended Kalman filter-based estimator. Specifically, we model the spoofer location $p_{s}$ as

(3) $$p_{s}\left(k\right)=p_{s}\left(k-1\right)+v_{s}\left(k-1\right)\Delta t$$

where $v_{s}$ is the velocity of the spoofer, and Δt is the time interval between the *k*-th and the (k−1)-th epochs. Following Ref. [24], the state vector is $x_{k}={\left[p_{s}{\left(k\right)}^{⊤},v_{s}{\left(k\right)}^{⊤},d^{us},a_{1},a_{2}\right]}^{⊤}$, where $d^{us}$, $a_{1}$, and $a_{2}$ define the coefficients in the quadratic polynomial method to estimate clock drift terms, leading to

(4) $$B_{f}\left(k\right)\approx L\left(k\right)+d^{us}+a_{1}k\Delta t+a_{2}{\left(k\Delta t\right)}^{2}+\xi \left(k\right)$$

where $\xi \left(k\right)∼N\left(0,R_{k}\right)$ is the observation noise. It is simplified as $\xi_{k}$ in the following observation model. Thus, we have the state transition model and measurement model as

(5) $$x_{k}=\Phi x_{k-1}+q_{k}$$

(6) $$y_{k}=h_{k}\left(x_{k}\right)+\xi_{k}.$$

Here, the nonlinear measurement function is

(7) $$h_{k}\left(x_{k}\right)=∥p_{s}\left(k\right)-p_{u}\left(k\right)∥+d^{us}+a_{1}k\Delta t+a_{2}{\left(k\Delta t\right)}^{2},$$

and the state transition matrix is $\Phi =\left[\begin{array}{ccc} I_{D} & \Delta t\;I_{D} & 0_{D\times 3}\\ 0_{D\times D} & I_{D} & 0_{D\times 3}\\ 0_{3\times D} & 0_{3\times D} & I_{3} \end{array}\right]\in R^{\left(2D+3)\times (2D+3\right)}$, where $I_{D}$ and $I_{3}$ denote the D×D and 3×3 identity matrices, respectively, and $0_{m\times n}$ denotes an m×n zero matrix. Here, *D* denotes the dimension of the spoofer position. $q_{k}∼N\left(0,Q_{k}\right)$ is the state transition noise, $H_{k}={\left.\frac{\partial h_{k}\left(x\right)}{\partial x}\right|}_{x=x_{k|k-1}}=\left[\frac{{\left({\hat{p}}_{s,k|k-1}-p_{u}\left(k\right)\right)}^{⊤}}{∥{\hat{p}}_{s,k|k-1}-p_{u}\left(k\right)∥},0_{1\times D},1,k\Delta t,{\left(k\Delta t\right)}^{2}\right]\in R^{1\times (2D+3)}$ is the Jacobian matrix of $h_{k}\left(x_{k|k-1}\right)$, and $y_{k}=B_{f}\left(k\right)$ is the observation. Furthermore, considering the uncertainty in the receiver position estimate, we reformulate the observation noise variance as

(8) $${\tilde{R}}_{k}=R_{k}+H_{k}P_{r}H_{k}^{⊤}$$

where $P_{r}=\mathrm{blkdiag}\left(P_{rD},\;0_{D\times D},\;0_{3\times 3}\right)\in R^{\left(2D+3)\times (2D+3\right)},$ with $P_{rD}$ representing the covariance of the receiver–position uncertainty. In the spoofer localization model, only the receiver–position estimate obtained from the receiver positioning solution at the same epoch is treated as an uncertain parameter. Other receiver solution states, such as the estimated receiver clock bias, are not explicitly introduced as parameters in the localization model in Ref. [4], and therefore the corresponding blocks in $P_{r}$ are set to zero.

Thus, the state vector containing the spoofer location, velocity, and clock-model parameters can be estimated using the standard extended Kalman filter algorithm:

(9) $$x_{k|k-1}=\Phi x_{k-1|k-1}$$

(10) $$P_{k|k-1}=\Phi P_{k-1|k-1}\Phi^{⊤}+Q_{k}$$

(11) $$K_{k}=P_{k|k-1}H_{k}^{⊤}{\left(H_{k}P_{k|k-1}H_{k}^{⊤}+{\tilde{R}}_{k}\right)}^{-1}$$

(12) $$x_{k|k}=x_{k|k-1}+K_{k}\left(y_{k}-h_{k}\left(x_{k|k-1}\right)\right)$$

(13) $$P_{k|k}=\left(I-K_{k}H_{k}\right)P_{k|k-1}$$

where $x_{k|k}$ is the estimated state and $P_{k|k}$ is the state-estimation error covariance approximation propagated by the EKF. The covariance will then be used to calculate the uncertainty used to assess the observation geometry quality.

Considering the fact that state initialization is required for the KF algorithm and the geometry information of the spoofer with respect to the receiver cannot be guaranteed to be valid, an LS estimator is first applied until the uncertainty calculated from $P_{k}$ in (16) falls below a certain threshold. The LS estimator is applied with a sliding window of *N* samples, assuming the spoofer velocity is constant over the sliding window:

(14) $${\hat{x}}_{k}^{\left(i\right)}={\hat{x}}_{k}^{\left(i-1\right)}+{\left({\tilde{H}}_{kN}^{⊤}{\tilde{H}}_{kN}\right)}^{-1}{\tilde{H}}_{kN}^{⊤}\left({\tilde{y}}_{kN}-{\tilde{h}}_{kN}\left({\hat{x}}_{k}^{\left(i-1\right)}\right)\right).$$

Here, ${\hat{x}}_{k}^{\left(i-1\right)}$ denotes the estimate obtained at iteration i−1 by the iterative LS estimator, ${\tilde{y}}_{kN}=\left[y_{k-N+1};y_{k-N+2};\cdots ;y_{k}\right]$ represents *N* clock bias observations used before the KF estimator is applied, ${\tilde{h}}_{kN}\left({\hat{x}}_{k}\right)=\left[\begin{array}{c} ∥{\hat{p}}_{s}^{k}\left(1\right)-p_{u}^{k}\left(1\right)∥+d^{us}+a_{1}\Delta_{1t}^{k}+a_{2}{\left(\Delta_{1t}^{k}\right)}^{2}\\ ∥{\hat{p}}_{s}^{k}\left(2\right)-p_{u}^{k}\left(2\right)∥+d^{us}+a_{1}\Delta_{2t}^{k}+a_{2}{\left(\Delta_{2t}^{k}\right)}^{2}\\ \vdots \\ ∥{\hat{p}}_{s}^{k}\left(N\right)-p_{u}^{k}\left(N\right)∥+d^{us}+a_{1}\Delta_{Nt}^{k}+a_{2}{\left(\Delta_{Nt}^{k}\right)}^{2} \end{array}\right]\in R^{N\times 1},$ and ${\tilde{H}}_{kN}$ is defined as

(15) $${\tilde{H}}_{kN}=\left[\begin{array}{ccccc} \frac{{\left[{\hat{p}}_{s}^{k}\left(1\right)-p_{u}^{k}\left(1\right)\right]}^{⊤}}{∥{\hat{p}}_{s}^{k}\left(1\right)-p_{u}^{k}\left(1\right)∥} & -\frac{{\left[{\hat{p}}_{s}^{k}\left(1\right)-p_{u}^{k}\left(1\right)\right]}^{⊤}}{∥{\hat{p}}_{s}^{k}\left(1\right)-p_{u}^{k}\left(1\right)∥}\left(N-1\right)\Delta t & 1 & \Delta_{1t}^{k} & {\left(\Delta_{1t}^{k}\right)}^{2}\\ \frac{{\left[{\hat{p}}_{s}^{k}\left(2\right)-p_{u}^{k}\left(2\right)\right]}^{⊤}}{∥{\hat{p}}_{s}^{k}\left(2\right)-p_{u}^{k}\left(2\right)∥} & -\frac{{\left[{\hat{p}}_{s}^{k}\left(2\right)-p_{u}^{k}\left(2\right)\right]}^{⊤}}{∥{\hat{p}}_{s}^{k}\left(2\right)-p_{u}^{k}\left(2\right)∥}\left(N-2\right)\Delta t & 1 & \Delta_{2t}^{k} & {\left(\Delta_{2t}^{k}\right)}^{2}\\ \ldots & \ldots & \ldots & \ldots & \ldots \\ \frac{{\left[{\hat{p}}_{s}^{k}\left(N\right)-p_{u}^{k}\left(N\right)\right]}^{⊤}}{∥{\hat{p}}_{s}^{k}\left(N\right)-p_{u}^{k}\left(N\right)∥} & 0_{1\times D} & 1 & \Delta_{Nt}^{k} & {\left(\Delta_{Nt}^{k}\right)}^{2} \end{array}\right],$$

where ${\hat{p}}_{s}^{k}\left(n\right)={\hat{p}}_{s}\left(k\right)-{\hat{v}}_{s}\left(k\right)\left(N-n\right)\Delta t$ denotes the predicted spoofer position at the *n*th epoch of the sliding window based on the current position and velocity under the constant-velocity model, $p_{u}^{k}\left(n\right)=p_{u}\left(k-N+n\right)$ is the corresponding receiver position, and $\Delta_{nt}^{k}=\left(k-N+n\right)\Delta t$ denotes the time instant corresponding to the *n*th observation in the sliding window. Furthermore, the corresponding covariance is estimated as

(16) $$P_{k}={\left({\tilde{H}}_{kN}^{⊤}{\tilde{H}}_{kN}\right)}^{-1}{\tilde{H}}_{kN}^{⊤}{\tilde{R}}_{kN}{\tilde{H}}_{kN}{\left({\tilde{H}}_{kN}^{⊤}{\tilde{H}}_{kN}\right)}^{-1}$$

To obtain ${\tilde{R}}_{kN}$, we start from the state-transition model in (5). Since the LS estimator uses the current state $x_{k}$ to represent the spoofer states associated with the previous observations in the window, we define the backward state-transition matrix as

(17) $$F=\Phi^{-1}.$$

Accordingly, the one-step backward transition can be written as

(18) $$x_{k-1}=Fx_{k}+{\bar{q}}_{k},$$

where

(19) $${\bar{q}}_{k}=-Fq_{k},\;{\bar{Q}}_{k}=\mathrm{Cov}\left({\bar{q}}_{k}\right)=FQ_{k}F^{⊤}.$$

Extending this relation to the state at epoch k−j gives

(20) $$x_{k-j}=F^{j}x_{k}+w_{k,j},$$

where the accumulated state-transition error is

(21) $$w_{k,j}=\sum_{l=0}^{j-1}F^{l}{\bar{q}}_{k-j+1+l}.$$

Assuming that the state-transition noises at different epochs are uncorrelated, the corresponding accumulated covariance is

(22) $$S_{k,j}=\mathrm{Cov}\left(w_{k,j}\right)=\sum_{l=0}^{j-1}F^{l}{\bar{Q}}_{k-j+1+l}{\left(F^{l}\right)}^{⊤},\;S_{k,0}=0.$$

For the *n*th observation in the sliding window, the corresponding epoch is k−N+n, with n=1,…,N, and its separation from the current epoch *k* is therefore $j_{n}=N-n.$ Thus, the process-noise contribution to the variance of the *n*th observation is

(23) $$r_{q,n}={\bar{H}}_{kN}^{\left(n\right)}S_{k,N-n}{\left({\bar{H}}_{kN}^{\left(n\right)}\right)}^{⊤},$$

where ${\bar{H}}_{kN}^{\left(n\right)}$ denotes the *n*th row of ${\bar{H}}_{kN}$, which is the Jacobian of the *n*th observation with respect to the state at its corresponding epoch k−N+n, evaluated at the backward-predicted state $F^{N-n}{\hat{x}}_{k}$. Unlike ${\tilde{H}}_{kN}^{\left(n\right)}$, which is the Jacobian with respect to the current state $x_{k}$ and therefore includes the effect of the current velocity on the backward-predicted position, ${\bar{H}}_{kN}^{\left(n\right)}$ has a zero velocity block because the observation at epoch k−N+n does not directly depend on the spoofer velocity. It is defined as

(24) $${\bar{H}}_{kN}=\left[\begin{array}{ccccc} \frac{{\left[{\hat{p}}_{s}^{k}\left(1\right)-p_{u}^{k}\left(1\right)\right]}^{⊤}}{∥{\hat{p}}_{s}^{k}\left(1\right)-p_{u}^{k}\left(1\right)∥} & 0_{1\times D} & 1 & \Delta_{1t}^{k} & {\left(\Delta_{1t}^{k}\right)}^{2}\\ \frac{{\left[{\hat{p}}_{s}^{k}\left(2\right)-p_{u}^{k}\left(2\right)\right]}^{⊤}}{∥{\hat{p}}_{s}^{k}\left(2\right)-p_{u}^{k}\left(2\right)∥} & 0_{1\times D} & 1 & \Delta_{2t}^{k} & {\left(\Delta_{2t}^{k}\right)}^{2}\\ \ldots & \ldots & \ldots & \ldots & \ldots \\ \frac{{\left[{\hat{p}}_{s}^{k}\left(N\right)-p_{u}^{k}\left(N\right)\right]}^{⊤}}{∥{\hat{p}}_{s}^{k}\left(N\right)-p_{u}^{k}\left(N\right)∥} & 0_{1\times D} & 1 & \Delta_{Nt}^{k} & {\left(\Delta_{Nt}^{k}\right)}^{2} \end{array}\right].$$

The two Jacobians are related through the backward state transition as

(25) $${\tilde{H}}_{kN}^{\left(n\right)}={\bar{H}}_{kN}^{\left(n\right)}F^{N-n}.$$

In particular, the observation at the current epoch does not require backward propagation and therefore $r_{q,N}=0.$

Accordingly, retaining the individual process-noise variances for the observations in the sliding window, the effective observation covariance is

(26) $${\tilde{R}}_{kN}=\mathrm{diag}\left[r_{q,1},r_{q,2},\ldots ,r_{q,N}\right]+R_{kN}+{\tilde{H}}_{kN}P_{r}{\tilde{H}}_{kN}^{⊤}.$$

where $R_{kN}=\mathrm{diag}\left[R_{k-N+1},R_{k-N+2},\ldots ,R_{k}\right]$. For simplicity, the receiver–position uncertainty is treated as a common error over the LS initialization window. Here, the dimensions of the quantities used in the sliding-window LS estimator are ${\hat{x}}_{k},x_{0}\in R^{(2D+3)\times 1}$, ${\tilde{H}}_{kN}\in R^{N\times (2D+3)},$ ${\tilde{y}}_{kN}\in R^{N\times 1}$, ${\tilde{R}}_{kN}\in R^{N\times N}$, and $P_{k}\in R^{\left(2D+3)\times (2D+3\right)}$.

Algorithm 1 presents pseudocode to show more details about the proposed method. Specifically, the LS estimator is first applied to obtain a rough estimation of the initial spoofer position and the corresponding uncertainty. Once the uncertainty calculated from the state estimation covariance $P_{k}$ falls below the specified threshold, the geometry is regarded as sufficient for accurate spoofer location filtering. Then the KF algorithm can be applied starting from the (k+1)-th epoch to update the initial estimate. In the simulations in this paper, the KF is initialized using the state mean and covariance both obtained from the LS estimate. The convergence threshold of the iterative LS estimator is set to $\epsilon_{\mathrm{LS}}=10^{-2}$, with a maximum of 100 iterations. For the dynamic-spoofer simulations, the process-noise covariance $Q_{k}$ is diagonal. Since the simulated spoofer trajectories follow the assumed constant-velocity model with only small random deviations, relatively small process-noise levels are used. The standard deviation of the position process noise is set to 0.1 m in both directions. For the velocity states, the standard deviation is set to 0.01 m/s along the dominant direction of motion and 0.001 m/s along the other direction, allowing small deviations from the constant-velocity assumption while preserving the overall motion pattern. Accordingly, the velocity process-noise standard deviations are 0.001 m/s and 0.01 m/s in the *x*- and *y*-directions for trajectory A, and 0.01 m/s and 0.001 m/s, respectively, for trajectory B. The process-noise variances of the three clock-model parameters are set to $10^{-20}$ because these polynomial parameters are assumed constant over the considered tracking interval; the small nonzero value is used only for numerical regularization. The same process-noise settings are used for the circular, square, and cross receiver trajectories.
**Algorithm 1:** Proposed algorithm
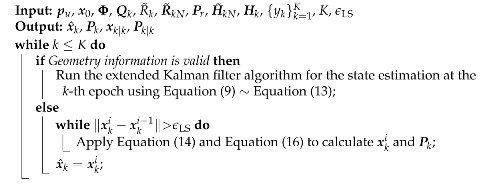


## 3. Simulation and Analysis

### 3.1. Static Spoofer Scenario

First we evaluate the localization performance of the proposed method compared to the one in Ref. [24]. Following Ref. [24], we include different receiver trajectories, including (1) circle, (2) square, and (3) cross. The position dimension is set to be D=2, without loss of generality. The considered trajectory extent is selected for controlled evaluation of the relative receiver–spoofer geometry. In practice, a larger search area may be required when the spoofer is farther away or when the available geometry is insufficient. The formulation can also be directly extended to three-dimensional receiver motion by setting D=3. Furthermore, we set the window size to N=100 and Δt=2 s. This corresponds to an observation window of 200 s. As illustrated in Ref. [24], based on the reported GNSS positioning performance [27,28], the standard deviation of the pseudorange measurement is assumed to be 3 m, which corresponds to a roughly 7 m standard deviation for the SPP fixes or equivalently a horizontal 95% circular error probability (CEP) of approximately 10 m. The receiver intercepts the spoofing signals to measure the counterfeit clock bias, the standard deviation of which is assumed to be 5 m, equivalent to an average timing-estimation accuracy of 50 nanoseconds.

For a receiver moving along a circular trajectory with a radius of r=1000 m, the receiver angular velocity is set to $v_{ra}=\frac{2\pi }{\Delta tN}=0.01\pi$ rad/s to complete a full circular trajectory within each sliding window, which means the linear velocity is $v_{r}=v_{ra}\;r=0.01\;\pi \;10^{3}=10\;\pi$ m/s, which is also consistent with Ref. [24] and is a common vehicle velocity.

Similarly, for a receiver moving along a square or a cross trajectory with a side length of L=2000 m, we set the receiver velocity $v_{r}=\frac{4L}{\Delta tN}=40$ m/s to form a complete trajectory for each sliding window.

Figure 1 presents the receiver trajectory (red line), the spoofer position (blue), the estimated spoofer positions obtained using Ref. [24] (green), and those obtained using the proposed method (magenta). From Figure 1, we can see that the proposed method provides more accurate spoofer localization than the LS estimator in all discussed scenarios. The same conclusion can be drawn from Table 1, where the Root Mean Square Error (RMSE) values of the position estimates are presented. This improvement is expected because the extended Kalman filter accumulates information from prior estimates. Furthermore, it can be observed that point C has a much smaller RMSE than points A and B for all three trajectories and for both LS and KF. This is because the observations at point C are obtained from substantially different directions, leading to more favorable geometry and improved localization accuracy.

### 3.2. Dynamic Spoofer Scenario

For a reasonable comparison, we keep the time interval Δt, sliding window size *N*, standard deviations of measurement errors, and receiver trajectory information the same as in the static spoofer scenario, while assigning two different trajectories to the spoofer: case A and case B, as shown in Figure 2. Specifically, the position of the spoofer is simulated based on a constant velocity model. For the purpose of simulating real-world variability and enhancing realism in the model, additional Gaussian noise with a standard deviation of 0.1 m is applied to the position at each epoch. As for the velocity, the current velocity is determined by the previous velocity plus Gaussian noise. Specifically, the spoofer in case A is initialized with velocity components of 0.5 m/s in the *x* direction and 4 m/s in the *y* direction; the spoofer in case B is initialized with velocity components of 4 m/s in the *x* direction and 0.5 m/s in the *y* direction. The standard deviations for the Gaussian noise added to the spoofer velocity are 0.001 m/s in the *x* direction and 0.01 m/s in the *y* direction for case A, and 0.01 m/s in the *x* direction and 0.001 m/s in the *y* direction for case B.

Figure 2 presents the trajectories of the receiver (red line) and the spoofer (blue), and the estimation of the spoofer position by the proposed method (green) over time. From Figure 2, we can observe that the proposed method works in all discussed scenarios, with the estimated spoofer trajectories following the true trajectories. Figure 3 and Figure 4 provide the positioning error in x/y coordinates (blue) and the uncertainty represented by the 3σ of the proposed method (red) in various scenarios, and the selected estimator (yellow) over time, where 0 indicates the LS estimator and 1 indicates the KF estimator. In Figure 3, we can see that the error and uncertainty decrease over time. This is because the spoofer moves closer to the receiver trajectory over time in case A, and the KF algorithm is applied after the Distance Root Mean Square (DRMS) falls below a threshold, which is set to 100 m in the simulation. Specifically, the spoofer generally moves toward the receiver trajectory in case A. As the distance decreases, the receiver positions within the estimation window provide more diverse relative observation directions, resulting in improved geometric observability. Therefore, both the estimation error and uncertainty generally decrease as the spoofer approaches the receiver trajectory. In Figure 4, the uncertainty increases significantly over time for all scenarios in the *x* coordinate, but only slightly in the *y* coordinate, because in case B the spoofer is moving further from the receiver horizontally compared to vertically. As the spoofer in case B moves predominantly away from the receiver trajectory along the *x* direction, the observation geometry gradually becomes less diverse in the radial direction. Consequently, the radial component of the localization state becomes less observable, which is reflected by the increasing *x* direction uncertainty. However, the errors remain relatively stable. This is because the KF is applied all the time in case B. With the accumulation of information from prior estimates by the KF, the current state estimation error does not increase significantly as the spoofer moves farther from the receiver. Furthermore, it should also be noted that the uncertainty represents the statistical confidence of the estimator rather than the instantaneous estimation error; therefore, the actual error in one realization does not necessarily follow the uncertainty curve exactly. To further compare the performance in different scenarios, we refer to Table 2, where the scenarios with square and circle trajectories have similar RMSE and DRMS values. The two values are calculated from errors between the position estimates and the true values, and from the square root of the trace of the position covariance in $P_{k}$, respectively, in both cases A and B. According to Table 2, the cross trajectory has the highest RMSE and DRMS. This is because the cross trajectory provides less diverse geometry information than the other two trajectories. Specifically, most receiver positions along the cross trajectory are concentrated along two directions. For an off-center spoofer, this results in less variation in the relative observation geometry and therefore weaker independent constraints on the spoofer position. Consequently, the localization solution becomes more sensitive to measurement errors, resulting in larger RMSE and DRMS values.

## 4. Conclusions

This paper extended the single-receiver spoofer localization method in Ref. [24] from static spoofer localization to dynamic spoofer tracking using an extended Kalman filter under a constant-velocity assumption. The proposed method retains the single-receiver and single-antenna advantages of the original method while achieving better localization accuracy than the original LS-based estimator for a static spoofer and enabling the tracking of a moving spoofer. This demonstrates the potential for implementation on mobile platforms, such as vehicle- or UAV-borne receivers, without requiring multiple spatially distributed and synchronized receivers.

This work could be further expanded by considering more general spoofer motion models beyond the constant-velocity assumption, especially for dynamic spoofers. Future work will also investigate scenarios involving multiple spoofers, evaluate the method using measured data and field experiments, and examine its robustness under more realistic propagation conditions such as multipath. Machine learning techniques may also be explored to improve the description of complex spoofer motion when sufficient additional information and training data are available.

## Figures and Tables

**Figure 1:**
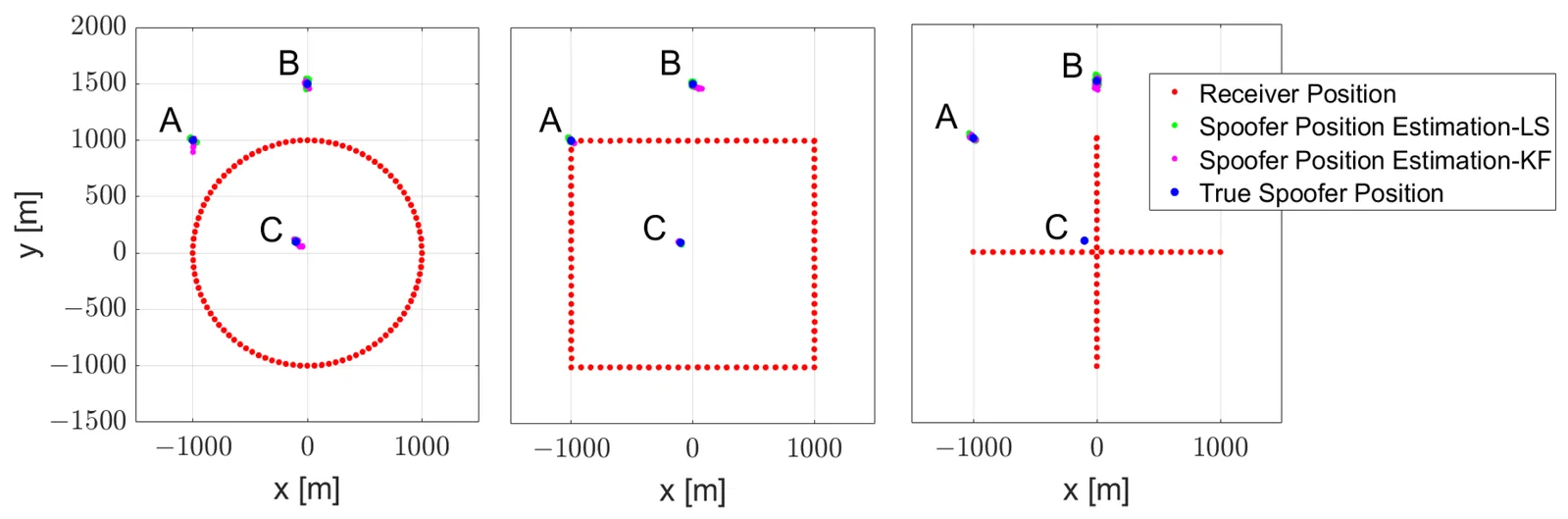
Performance of the proposed method and the LS estimator [24] for circular (**left**), square (**middle**), and cross (**right**) receiver trajectories, with the spoofer located at positions A, B, and C, where the red line shows the receiver trajectory, the blue point represents the true spoofer position, the green points indicate the corresponding estimates obtained by the LS estimator [24] and the magenta points show the estimates by the proposed method.

**Figure 2:**
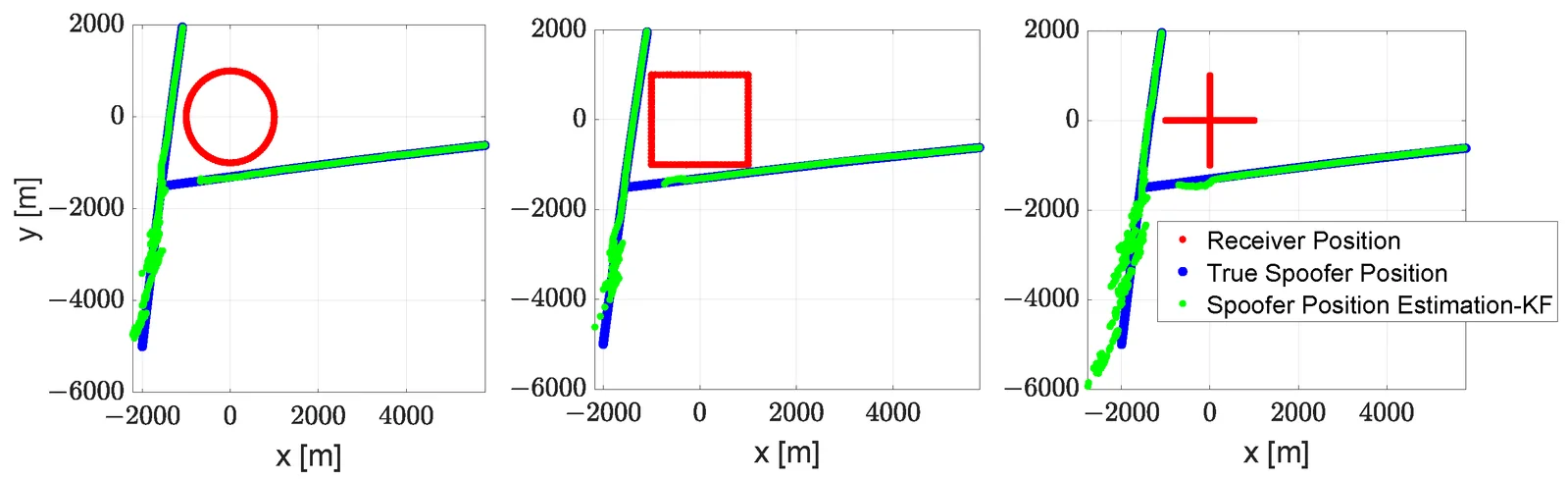
Performance of the proposed method for circular (**left**), square (**middle**), and cross (**right**) receiver trajectories, with the spoofer following trajectories A and B, where the red line shows the trajectory of the receiver, the blue line represents the true spoofer trajectory (both A and B), and the green line indicates the corresponding estimate by the proposed method.

**Figure 3:**
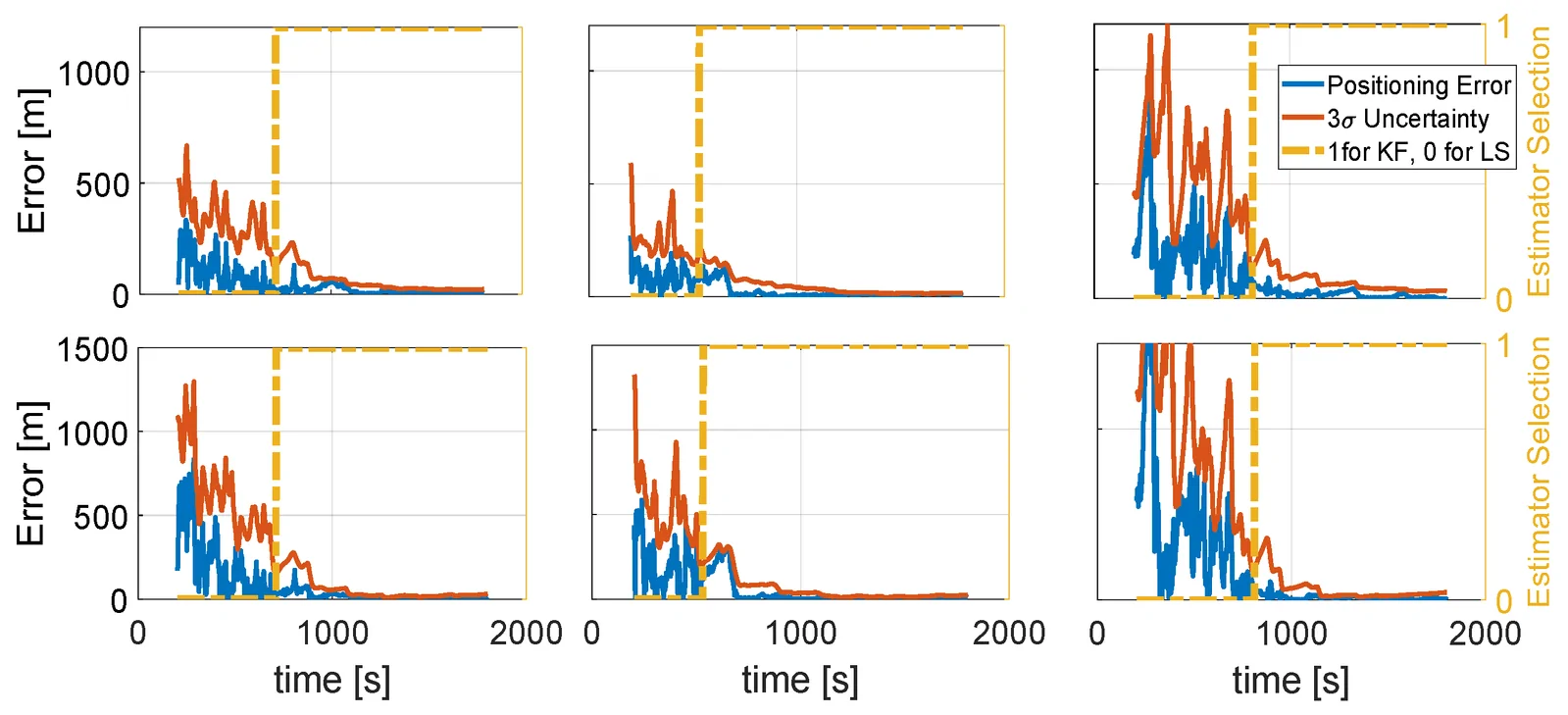
Performance of the proposed method in case A for circular (**left**), square (**middle**), and cross (**right**) receiver trajectories, where the red line shows uncertainty calculated as 3σ of the proposed method, the blue line represents the actual estimation error, and the yellow line indicates the selected estimator. The estimator is switched from LS to KF when the DRMS falls below the 100 m threshold, where 0 denotes the LS estimator and 1 denotes the KF estimator. The upper figures show the positioning error in *x* coordinate and the lower ones show the positioning error in *y* coordinate.

**Figure 4:**
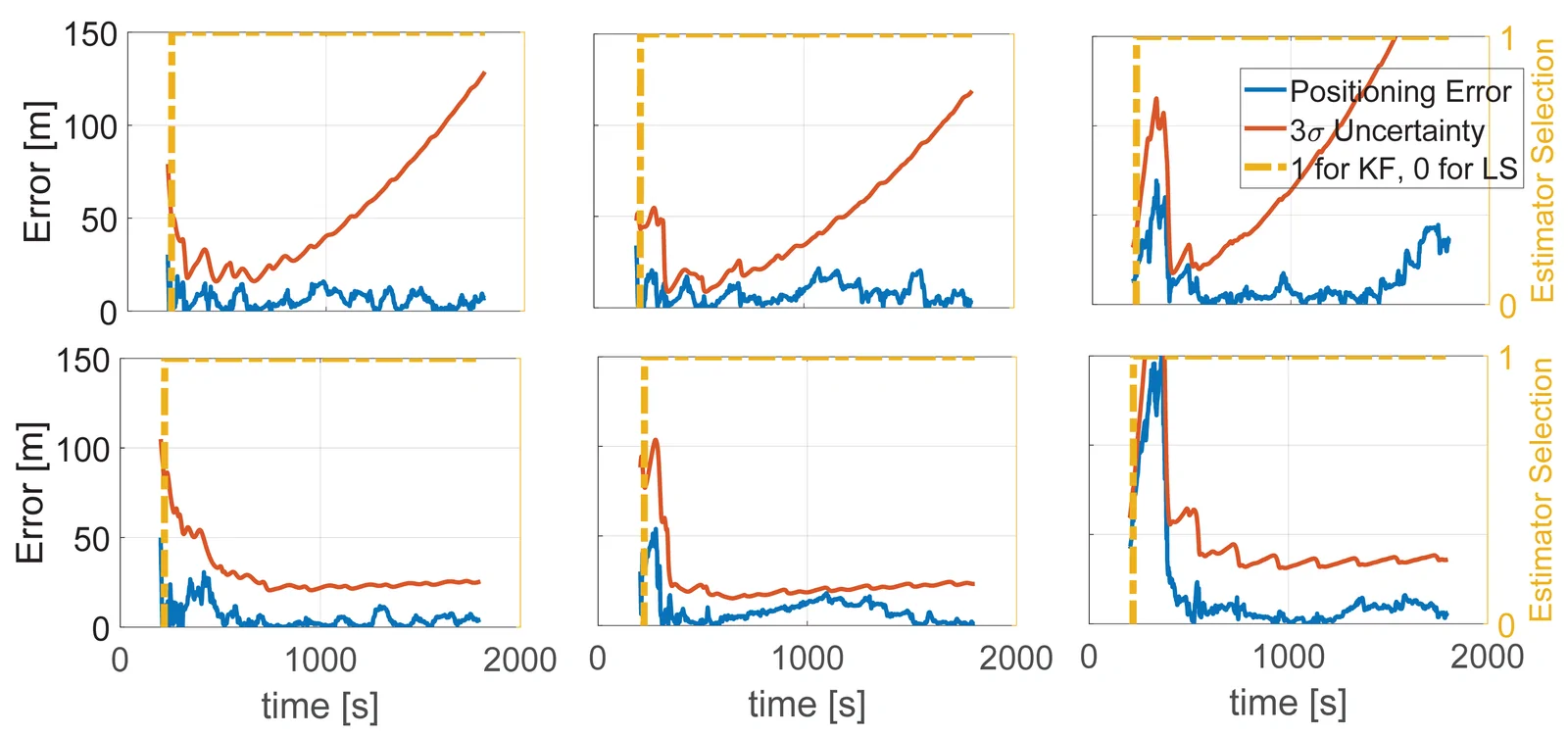
Performance of the proposed method in case B for circular (**left**), square (**middle**), and cross (**right**) receiver trajectories, where the red line shows uncertainty calculated as 3σ of the proposed method, the blue line represents the actual estimation error, and the yellow line indicates the selected estimator. The estimator is switched from LS to KF when the DRMS falls below the 100 m threshold, where 0 denotes the LS estimator and 1 denotes the KF estimator. The upper figures show the positioning error in *x* coordinate and the lower ones show the positioning error in *y* coordinate.

**Table 1:** RMSE of the proposed KF estimator and the comparison method under different receiver trajectories and spoofer positions.

Receiver	Circle			Square			Cross		
Spoofer	A	B	C	A	B	C	A	B	C
RMSE (LS) [m]	6.70	9.50	2.94	6.52	4.78	2.78	7.82	11.87	1.72
RMSE (KF) [m]	3.30	4.21	0.99	3.26	1.48	0.92	3.22	6.47	0.83

**Table 2:** RMSE and DRMS of the proposed KF estimator under different receiver and spoofer trajectories.

Receiver	Circle		Square		Cross	
Spoofer	A	B	A	B	A	B
RMSE [m]	63.84	6.40	52.17	9.01	133.86	18.41
DRMS [m]	92.60	21.75	60.12	19.28	166.84	36.09

## Data Availability

The simulated test is designed based on the parameters reported in Ref. [24].

## References

[1] Psiaki M.L., Humphreys T.E. (2016). GNSS spoofing and detection. Proc. IEEE.

[2] Humphreys T.E., Ledvina B.M., Psiaki M.L., O’Hanlon B.W., Kintner P.M. Assessing the spoofing threat: Development of a portable GPS civilian spoofer. Proceedings of the 21st International Technical Meeting of the Satellite Division of the Institute of Navigation (ION GNSS 2008).

[3] Montgomery P.Y., Humphreys T.E., Ledvina B.M. Receiver-autonomous spoofing detection: Experimental results of a multi-antenna receiver defense against a portable civil GPS spoofer. Proceedings of the 2009 International Technical Meeting of The Institute of Navigation.

[4] Akos D.M. (2012). Who’s afraid of the spoofer? GPS/GNSS spoofing detection via automatic gain control (AGC). Navig. J. Inst. Navig..

[5] Broumandan A., Jafarnia-Jahromi A., Dehghanian V., Nielsen J., Lachapelle G. (2012). GNSS spoofing detection in handheld receivers based on signal spatial correlation. Proceedings of the 2012 IEEE/ION Position, Location and Navigation Symposium.

[6] Zhang G., Zhang Y., Tian Y. (2019). Research of Beidou navigation satellite system spoofing detection based on DOD and PTD. Appl. Sci. Technol..

[7] Dehghanian V., Nielsen J., Lachapelle G. GNSS spoofing detection based on receiver C/No estimates. Proceedings of the 25th International Technical Meeting of the Satellite Division of the Institute of Navigation (ION GNSS 2012).

[8] Liu J., Chen F., Xie Y., Ge B., Lu Z., Sun G. (2023). Robust spoofing detection for GNSS array instrumentation based on C/N0 difference measurements. IEEE Trans. Instrum. Meas..

[9] Hegarty C., O’Hanlon B., Odeh A., Shallberg K., Flake J. Spoofing detection in GNSS receivers through cross-ambiguity function monitoring. Proceedings of the 32nd International Technical Meeting of the Satellite Division of the Institute of Navigation (ION GNSS+ 2019).

[10] Ahmed S., Khanafseh S., Pervan B. GNSS spoofing detection based on decomposition of the complex cross ambiguity function. Proceedings of the 34th International Technical Meeting of the Satellite Division of the Institute of Navigation (ION GNSS+ 2021).

[11] Borhani-Darian P., Li H., Wu P., Closas P. Deep neural network approach to detect GNSS spoofing attacks. Proceedings of the 33rd International Technical Meeting of the Satellite Division of the Institute of Navigation (ION GNSS+ 2020).

[12] Borhani-Darian P., Li H., Wu P., Closas P. (2024). Detecting GNSS spoofing using deep learning. Eurasip J. Adv. Signal Process..

[13] Shang X., Sun F., Zhang L., Cui J., Zhang Y. (2022). Detection and mitigation of GNSS spoofing via the pseudorange difference between epochs in a multicorrelator receiver. GPS Solut..

[14] Radin D., Swaszek P.F., Seals K.C., Hartnett R.J. GNSS spoof detection based on pseudoranges from multiple receivers. Proceedings of the 2015 International Technical Meeting of the Institute of Navigation.

[15] Demir M.Ö., Kurt G.K., Pusane A.E. (2023). A pseudorange-based GPS spoofing detection using hyperbola equations. IEEE Trans. Veh. Technol..

[16] Dinh T.N., Huu T.T., Van H.N., La The V., Ngoc H.P., Hai T.T., Hoang-Van H. (2024). A method to build feature descriptor for GNSS spoofing detection by carrier phase double difference measurement. Meas. Sci. Technol..

[17] Psiaki M.L., O’Hanlon B.W., Powell S.P., Bhatti J.A., Wesson K.D., Humphreys T.E., Schofield A. GNSS spoofing detection using two-antenna differential carrier phase. Proceedings of the 27th International Technical Meeting of the Satellite Division of the Institute of Navigation (ION GNSS+ 2014).

[18] Chen Z., Li J., Li J., Zhu X., Li C. (2022). GNSS multiparameter spoofing detection method based on support vector machine. IEEE Sens. J..

[19] Chen Y., Trappe W., Martin R.P. (2007). Detecting and localizing wireless spoofing attacks. Proceedings of the 2007 4th Annual IEEE Communications Society Conference on Sensor, Mesh and ad Hoc Communications and Networks.

[20] Tomic S., Beko M., Camarinha-Matos L.M., Oliveira L.B. (2019). Distributed localization with complemented RSS and AOA measurements: Theory and methods. Appl. Sci..

[21] Bhatti J.A., Humphreys T.E., Ledvina B.M. (2012). Development and demonstration of a TDOA-based GNSS interference signal localization system. Proceedings of the 2012 IEEE/ION Position, Location and Navigation Symposium.

[22] Jansen K., Tippenhauer N.O., Pöpper C. Multi-receiver GPS spoofing detection: Error models and realization. Proceedings of the 32nd Annual Conference on Computer Security Applications.

[23] Chen W., Dong Y., Duan Z. (2024). Impacts of a Single GPS Spoofer on Multiple Receivers: Formal Analysis and Experimental Evaluation. Proceedings of the 2024 IEEE 21st Consumer Communications & Networking Conference (CCNC).

[24] Chen X., Morton Y., Yu W.X., Truong T.K. (2023). GNSS Spoofer Localization with Counterfeit Clock Bias Observables on a Mobile Platform. IEEE Sens. J..

[25] Clements Z., Goodridge I., Ellis P., Murrian M.J., Humphreys T.E. Demonstration of Single-Satellite GNSS Spoofer Geolocation. Proceedings of the 2024 International Technical Meeting of the Institute of Navigation.

[26] Friedland B., Bernstein I. (1966). Estimation of the State of a Nonlinear Process in the Presence of Nongaussian Noise and Disturbances. J. Frankl. Inst..

[27] Groves P. (2013). Principles of GNSS, Inertial, and Multisensor Integrated Navigation Systems.

[28] U.S. Department of Defense (2020). Global Positioning System Standard Positioning Service Performance Standard.

